# Transfer Entropy Reconstruction and Labeling of Neuronal Connections from Simulated Calcium Imaging

**DOI:** 10.1371/journal.pone.0098842

**Published:** 2014-06-06

**Authors:** Javier G. Orlandi, Olav Stetter, Jordi Soriano, Theo Geisel, Demian Battaglia

**Affiliations:** 1 Departament d'Estructura i Consituents de la Matèria, Universitat de Barcelona, Barcelona, Spain; 2 Max Planck Institute for Dynamics and Self-Organization, Göttingen, Germany; 3 Georg-August-Universität, Physics Department, Göttingen, Germany; 4 Bernstein Center for Computational Neuroscience, Göttingen, Germany; 5 Institut de Neurosciences des Systèmes, Inserm UMR1106, Aix-Marseille Université, Marseille, France; Universitat Pompeu Fabra, Spain

## Abstract

Neuronal dynamics are fundamentally constrained by the underlying structural network architecture, yet much of the details of this synaptic connectivity are still unknown even in neuronal cultures *in vitro*. Here we extend a previous approach based on information theory, the Generalized Transfer Entropy, to the reconstruction of connectivity of simulated neuronal networks of both excitatory and inhibitory neurons. We show that, due to the model-free nature of the developed measure, both kinds of connections can be reliably inferred if the average firing rate between synchronous burst events exceeds a small minimum frequency. Furthermore, we suggest, based on systematic simulations, that even lower spontaneous inter-burst rates could be raised to meet the requirements of our reconstruction algorithm by applying a weak spatially homogeneous stimulation to the entire network. By combining multiple recordings of the same *in silico* network before and after pharmacologically blocking inhibitory synaptic transmission, we show then how it becomes possible to infer with high confidence the excitatory or inhibitory nature of each individual neuron.

## Introduction

Important advances in the last decade have provided unprecedented detail on the structure and function of brain circuits [1], [2] and even programs aiming at an exhaustive mapping of the brain connectome(s) have been announced [3]–[6]. First, the combination of invasive and non-invasive techniques such as high-resolution optical imagery and diffusion-based tractography have revealed the major architectural traits of brain circuitry [7]. Second, functional imaging has provided non-invasive measures of brain activity, both at rest [8] and during the realization of specific tasks [2]. These efforts have opened new perspectives in neuroscience and psychiatry, for instance to identify general principles underlying interactions between multi-scale brain circuits [9], [10], to probe the implementation of complex cognitive processes [11], [12], and to design novel clinical prognosis tools by linking brain pathologies with specific alterations of connectivity and function [13]–[15]. At the same time, tremendous technological advancements in serial-section electron microscopy are making the systematic investigation of synaptic connectivity at the level of detail of cortical microcircuits accessible [16].

Despite continuous progresses, the understanding of inter-relations between the observed functional couplings and the underlying neuronal dynamics and circuit structure is still a major open problem. Several works have shown that functional connectivity [17] at multiple scales is reminiscent of the underlying structural architecture [8], [18], [19]. This structure-to-function correspondence is, however, not direct and is rather mediated by interaction dynamics. On one side (“functional multiplicity”), structural networks generating a large reservoir of possible dynamical states can give rise to flexible switching between multiple functional connectivity networks [20], [21]. On the other (“structural degeneracy”), very different structural networks giving rise to analogous dynamical regimes may generate qualitatively similar functional networks [22]. Therefore, particular care is required when interpreting data originating from non-invasive functional data-gathering approaches such as fMRI [23]. Altogether, these arguments call for highly controllable experimental frameworks in which the results and predictions of different functional connectivity analysis techniques can be reliably tested in different dynamic regimes.

A first step in this endeavor consists in simplifying the neuronal system under investigation. For this reason, different studies have focused on *in vitro* neuronal cultures of dissociated neurons [24], [25]. Neuronal cultures are highly versatile and easily accessible in the laboratory. Unlike in naturally formed neuronal tissues, the structural connectivity in cultures can be dictated to some extent [25], and even neuronal dynamical processes can be regulated using pharmacological agents or optical or electrical stimulation. These features have made neuronal cultures particularly attractive for unveiling the processes shaping spontaneous activity, including its initiation [26], [27], synchronization [28] and plasticity [29], [30], as well as self-organization [31] and criticality [32]. Moreover, some studies also showed that spontaneous activity in *in vitro* preparations shares several dynamical traits with the native, naturally formed neuronal tissues [33].

A second step consists in developing and testing the analysis tools that identify directed functional interactions between the elements in the network. Information theoretic measures such as Transfer Entropy (TE) [34], [35] can capture linear and non-linear interactions between any pair of neurons in the network. TE does not require any specific interaction model between the elements, and therefore it is attracting a growing interest as a tool for investigating functional connectivity in imaging or electrophysiological studies [36]–[39]. The independence of TE on assumptions about interaction models has made it adequate to deal with different neuronal data, typically spike trains from simulated networks [40], multi-electrode recordings [41]–[44] or calcium imaging fluorescence data [22]. TE proved to be successful in describing topological features of functional cortical cultures [41], [42], [44], and in reconstructing structural network connectivity from activity [22], [43].

In a previous work [22], we investigated the assessment of excitatory-only structural connectivity from neuronal activity data (with inhibitory synaptic transmission blocked). For this purpose we developed an extension of TE, termed Generalized Transfer Entropy (GTE). To test the accuracy of our connectivity reconstruction method, we considered realistic computational models that mimicked the characteristically bursting dynamics of spontaneously active neuronal cultures. Comparing diverse reconstruction approaches, we concluded that GTE performed superiorly, even when systematic artifacts such as light scattering were explicitly added to our surrogate data. Besides the inclusion of corrections coping with the poor temporal resolution of typical calcium fluorescence recordings, a key ingredient making GTE successful was dynamical state selection, i.e. the restriction of the analysis to a dynamical regime in which functional interactions were largely determined by the underlying hidden structural connectivity. In particular we showed that it was necessary to restrict the analysis to inter-burst regimes, while consideration of bursting epochs led to inference of exceedingly clustered structural topologies [22].

Here we extend our previous work, by attempting the inference of both excitatory and inhibitory connectivity. Inhibition is a major player in regulating neuronal network dynamics, and the regulation of the excitatory-inhibitory balance is crucial for optimal circuit function [45], [46]. In the brain, inhibition shapes cortical activity [47], dominates sensory responses [48], and regulates motor behavior [49]. Severe behavioral deficits in psychiatric diseases such as autism and schizophrenia have been ascribed to an imbalance of the excitatory and inhibitory circuitry [50]. Despite the importance of inhibition, functional connectivity studies often disregard it because of the difficulty in its identification. Hence, unraveling inhibitory connections, and their interplay with the excitatory ones in shaping network dynamics, is of major interest. We show here that the TE-based approach that we previously used for the inference of excitatory connectivity can be extended with virtually no modifications to networks including as well inhibitory interaction, whose dynamics is once again reproduced by realistic computational models for which the ground-truth connectivity is known. We reveal that the most difficult inference problem is not the identification of a link, be it excitatory or inhibitory, but rather the correct labeling of its type. We show that an elevated accuracy of labeling of both excitatory and inhibitory links can be obtained by combining the analysis of network activity in two conditions, a first one where both excitation and inhibition are active, and a second one where inhibition is pharmacologically removed. We show as well, however, that the inference of link types remain extremely uncertain with current experimental protocols. As a perspective solution, we foresee, based on extensive simulations, that significant improvements in both reconstruction and labeling performance could be achieved by enhancing the spontaneous firing of a culture through a weak external stimulation.

## Results

### Dynamics of biological and simulated networks

**Figure 1:**
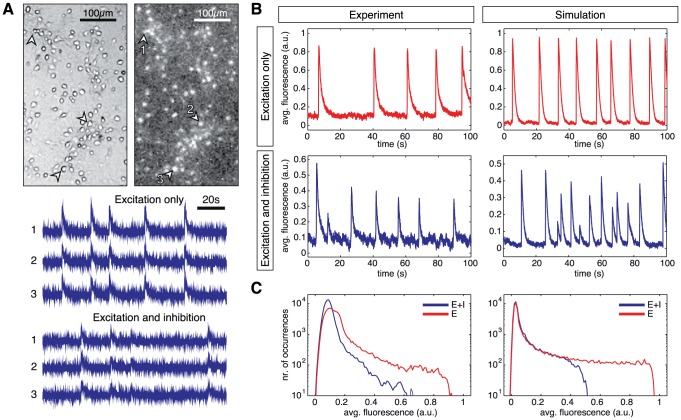
Neuronal network dynamics. **A** Top: Bright field and fluorescence images of a small region of a neuronal culture at day *in vitro* 12. Bright spots correspond to firing neurons. Bottom: Representative time traces of recorded fluorescence signals of 3 individual neurons. The numbers beside each trace identify the neurons on the images. Data shows, for the same neurons, the signal in recordings with only excitation active (“E”) and the signal with both excitation and inhibition active (“E+I”). **B** Population-averaged fluorescence signals in experiments (left) and simulations (right), illustrating the semi-quantitative matching between *in vitro* and *in silico* data. Top: excitatory-only traces (“E–only” data). For the experiments, inhibition was silenced through application of saturating concentrations of bicuculline. For the simulations, inhibitory synapses were silenced by setting their efficacy to zero. Bottom: traces for both excitation and inhibition active (“E+I” data). Network bursts appear as a fast increase of the fluorescence signal followed by a slow decay. Bursts are more frequent and display lower and more heterogeneous amplitudes in the presence of inhibitory connections. **C** Histogram of population-averaged fluorescence intensity for a 1 h recordings in experiments (left) and simulations (right). Data is shown in semilogarithmic scale for clarity. Red curves correspond to the “E–only” condition, and the blue curves to the “E+I” one.

Dissociated neurons grown *in vitro* self-organize and connect to one another, giving rise to a spontaneously active neuronal network within a week (see Figure 1A) [24], [30], [51], [52]. About 70–80% of the grown connections are excitatory, while the remaining 20–30% are inhibitory [51]. Activity in neuronal cultures is characterized by a bursting dynamics, where the whole network is active and displays quasi-synchronous, high frequency firing within 100–200 ms windows [30]. The timing of the bursts themselves is irregular, with average inter-burst intervals on the order of 10 s in a typical preparation. Between different bursts, firing across the network has a low-frequency and can be described as asynchronous.

Neuronal dynamics in cultures may be monitored using calcium fluorescence imaging (see Methods)[24], [53], which enables the recording of the activity of thousands of individual neurons simultaneously. Figure 1A shows example traces illustrating the characteristic fluorescence signal of individual neurons *in vitro*. The fluorescence signal is characterized by a fast onset as a result of neuronal activation and the binding of 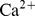 ions to the fluorescence probe, followed by a slow decay back to the baseline due to the slow unbinding rate. This behavior is apparent in the population average of the signal, as shown in Fig. 1B, where bursts are clearly identified by the fast rise of the fluorescence signal.

To appraise the role of inhibition on dynamics, we monitor neuronal network activity in two different conditions: A first one, with only excitatory connections active, where inhibitory connections have been completely blocked (denoted as “E–only” networks); and a second one, where both excitatory and inhibitory connections are functionally active (herein after denoted as “E+I” networks). In experiments, inhibitory synapses are silenced through the application of saturating levels of bicuculline, a GABA_A_ receptor antagonist (see Methods). An example trace of the population average signal of such an excitatory-only system is shown in the top left panel of Fig. 1B, whereas the dynamic behavior in presence of inhibition is shown in the bottom left panel of Fig. 1B. In the “E–only” condition, bursts are more pronounced and more regular in amplitude than in the “E+I” condition, an effect also seen in other studies [30], [54], [55].

These recordings in neuronal cultures provide a comparison reference for our simulated networks of model neurons. We build a computational model of a culture whose dynamics capture its major qualitative features. These include a high variability in the inter-burst intervals, a low 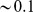 Hz inter-burst firing rate, and, in presence of inhibition, an increase in bursting frequency as well as a general decay in the amplitudes of the fluorescence signal, paired by an increase in their heterogeneity. More specifically, we consider a network of 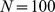 leaky integrate-and-fire nodes with depressive synapses in combination with a model for the calcium fluorescence. Network connectivity is random and sparse, with links rewired in order to reach an above-chance level of clustering (see Methods). Each node receives inputs from its pre-synaptic neighbors as well as from independent external sources to mimic spontaneous single neuron activity due to noise fluctuations in the ionic current through its membrane. Free model parameters, such as the homogeneous conductance weights of recurrent connections, were calibrated such as to yield dynamics comparable to the biological recordings, with a bursting rate of 0.1 Hz and realistic decay time constants of the calcium fluorescence (see the bottom right panels of Figure 1B). The blocking of inhibitory connections (top right panel of Figure 1B) is simulated by setting the synaptic weight of all inhibitory connections to zero (note, therefore, that the firing itself of inhibitory neurons is not suppressed, but just its postsynaptic effects).

As discussed more in depth in [22], a hallmark of bursting dynamics is the right-skewed histogram of the population average of the calcium fluorescence signal (see Figure 1C). Low fluorescence amplitudes are associated to the non-bursting regime, which is noise dominated, and the right tail of the distribution reflects bursting events. The range spanned by this right tail is distinctly shortened in presence of inhibition. This difference in the large fluorescence amplitude distribution can be ascribed to the dynamics at the synapse level: For purely excitatory networks, the neurotransmitters resources of a given synapse are depleted during a bursting event [56]. Neurons experience high frequency discharge, but require a longer time to recover, giving rise to long inter-burst intervals. Inhibition lowers this release of neurotransmitters by suppressing neuronal firing before complete depletion, therefore providing a faster recovery, shorter inter-burst periods and lower firing activity inside the bursts.

### Reconstructing structural connectivity from directed functional links

Based on simulations of the calcium dynamics in the network, a network of (directed) functional connectivity is reconstructed by computing the Generalized Transfer Entropy (GTE) for each (directed) pair of links (see Methods). GTE is an extension of Transfer Entropy, a measure that quantifies predictive information flow between stationary systems evolving in time [35]. As an information theoretical implementation of the Granger Causality concept [57], a positive TE score assigned to a directed link from a neuron 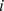 to a neuron 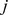 indicates that the future fluorescence of 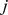 can be better predicted when considering as well the past fluorescence of 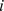 in addition to the past of 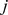 itself. We previously introduced GTE to study the reconstructed topology of purely excitatory networks under diverse network dynamical states and signal artifacts [22]. Here we extend its applicability to data that includes inhibitory action.

#### Conditioning as state selection

A central observation that motivated the definition of GTE was the existence of different dynamical states in the switching behavior from asynchronous firing to synchronous bursting activity. The distribution of fluorescence amplitudes (see Figure 1C) provides a visual guide to the relative weight of the single activity events and the bursting episodes. A functional reconstruction in this bursting regime shows a very clustered connectivity due to the tightly synchronized firing of large communities of neurons. We can understand intuitively this finding, by considering that, in the bursting regime, the network is over-excitable and the firing of a single neuron can trigger the firing of a large number of other neurons not necessarily linked to it by a direct synaptic link. On the other hand, the neuronal activity in the non-bursting regime is sparse and dominated by pairwise interactions, and thus, a reconstruction in this regime identifies directed functional interactions that more closely match the structural connectivity (i.e. high GTE might signal direct pre- to post-synaptic coupling in this regime), as previously discussed thoroughly for “E–only” networks [22].

**Figure 2:**
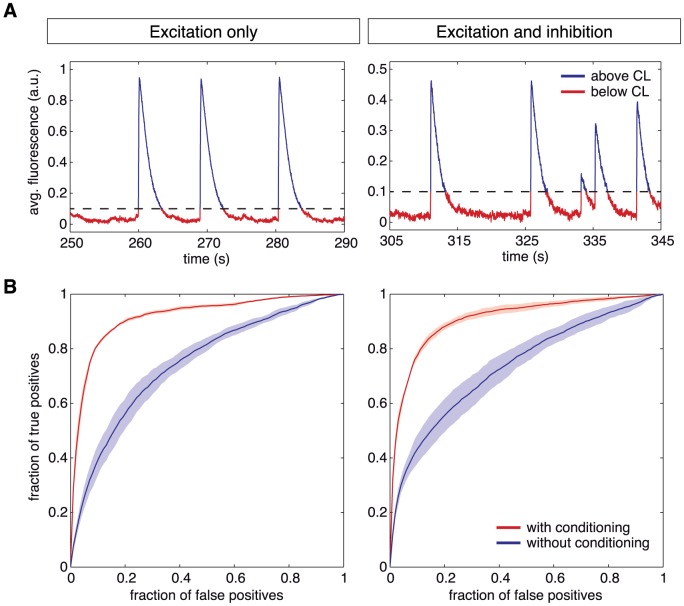
Signal conditioning. **A** Separation of the signal in two regimes according to the conditioning level (dotted line), a first one that encompasses the low activity events (red curves), and a second one that includes the bursting regimes only (blue). The same conditioning procedure is applied in both “E–only” networks (left) and in “E+I” ones (right). **B** *Receiver Operating Characteristic* (ROC) curves quantify the accuracy of reconstruction and its sensitivity on conditioning. Functional networks are generated by including links with a calculated GTE score exceeding an arbitrary threshold. ROC curves plot then the fraction of true and false positives in the functional networks inferred for every possible threshold. For “E–only” networks (left) and “E+I” networks (right), the red curves show the goodness of the reconstruction after applying the conditioning procedure. Blue curves illustrate the reconstruction performance without conditioning. The ROC curves show that the conditioning procedure significantly improves reconstruction performance. ROC curves were averaged over different network realizations (95% confidence intervals shown).

A rough segmentation of the population signal into time sequences of bursting and non-bursting events is simply achieved by defining a fixed *conditioning level* on the population average fluorescence. This simple modification with respect to the original TE formulation, makes GTE suitable for an analysis of functional interactions which distinguish different dynamical regimes, as illustrated for purely excitatory networks in the left panel of Figure 2A. The network is indeed considered to be in a bursting regime when the network-averaged fluorescence exceeds the chosen conditioning level (dotted line in Figure 2A), and in an inter-burst regime otherwise. The value of the conditioning level itself is obtained through the analysis of the fluorescence signal histogram and set close to the transition from the Gaussian-like profile shown for low fluorescence values to the long tail characteristic of the population bursts.

Note that, while our approach works by restricting the analysis to epochs of inter-burst activity only, other complementary methods exploit detailed information about typical burst build-up sequences in order to infer structure, with potentially superior results when the required time resolution is accessible (e.g. [58]).

#### Connectivity reconstruction of simulated “E–only” networks

Reconstruction performances from the GTE computation are quantified in the form of *receiver operating characteristic* (ROC) curves. These curves are obtained as follows: GTE assigns a score to every possible link in the network, and only scores above a given threshold are considered as putative links. These accepted links are then systematically compared with the ground truth topology of the network, and for gradually lower threshold levels. The ROC curves finally plot the fraction of true positives, i.e., inferred connections which really exist, as a function of the fraction of false positives, i.e., wrongly inferred connections.

The ROC curves of the reconstruction performance, with and without conditioning, for the case of simulated “E–only” networks are shown in the left panel of Figure 2B. Without conditioning (blue ROC curves), the reconstruction quality of excitatory connections — to both excitatory and inhibitory neurons confounded — is significantly better than a random choice (which would correspond to a diagonal line in the ROC curve). The reconstruction is, however, hindered by the fact that the analysis effectively averages over data from multiple dynamical regimes as described above. The reconstruction performance thus significantly increases by applying a conditioning (red ROC curves) which selects uniquely the inter-burst regime.

It was also shown for simulations comparable to the ones generated as described above, that the reconstructed networks using GTE are approximately unbiased regarding bulk network properties, such as the mean clustering coefficient, or the average length of connections in the network [22].

#### Connectivity reconstruction of simulated “E+I” networks

An important aspect of Transfer Entropy, and by extension of GTE, is its model-free nature. Thus, during the process of identifying causal influences between neurons, there is no need to define a generative model for neuronal firing or calcium dynamics, as in the case, e.g., of Bayesian inference approaches [59]. It follows that we can apply GTE without modifications to the case in which both excitatory and inhibitory links are active, provided that the inter-burst network state can be identified in an analogous way. Indeed we observe that while the presence of inhibition does change the dynamics of the system to some extent, the switching behavior remains robustly present (see the right panel of Figure 2A), allowing the straightforward identification of a performing conditioning level.

Remarkably, the reconstruction performance of “E+I” networks remains at high levels after conditioning, of about 80% true positives at 10% false positives, as shown in the right panel of Figure 2B. Thus the model-independence of GTE allows the reconstruction of both excitatory and inhibitory links. As a further self-consistency check, we have simulated the dynamics of a neuronal culture with a topology identical to the inferred one and compared it with the dynamics of the network with the original ground-truth topology. The resulting bursting and firing rates, for both the “E-only” and the “E+I” cases, are not statistically significantly different from the case of perfect reconstruction, while they markedly differ from the case of a randomized topology (not shown). Nevertheless, given the phenomenon of structural degeneracy, a large number of even very different structural circuits could give rise to equivalent dynamical regimes [22]. Therefore, passing this self-consistency check is not a sufficient condition to prove high reconstruction quality, though it is a necessary one.

Note, finally, that we have disregarded, until now, the identification of the specific type, i.e. excitatory or inhibitory, of each link, focusing uniquely on whether a link is present or absent in the ground-truth structural network, whatever is its nature. As previously mentioned, correctly labeling a link turns out to be a more elaborated task than just inferring its existence.

### Distinguishing excitatory and inhibitory links

GTE probes the existence of unspecified influences between signals, but cannot identify the type of occurring interaction *a priori*. Its versatility also means that very different types of interactions can give the same GTE score if their influence in terms of predictability is the same. Hence, to separate between excitatory and inhibitory connections we have to either introduce *ad hoc* information on neuronal types or combine different reconstructions together to single out the different connectivity types.

Such *ad hoc* information might come from dye impregnation, fluorescence labeling or immunostaining [60]. These techniques identify cell bodies and processes according to some specific traits, for instance membrane proteins or neurotransmitters' receptors. According to Dale's principle [61], a neuron shows the same distribution of neurotransmitters along its presynaptic terminals. Hence, if a neuron is labeled as either excitatory or inhibitory, we can assume that all its output connections are of the same matching type. Thus by combining the type of information provided by some extrinsic labeling technique with the unspecific causal information provided by GTE, the overall set of inferred links can be separated into two non–overlapping subsets of excitatory and inhibitory links.

**Figure 3:**
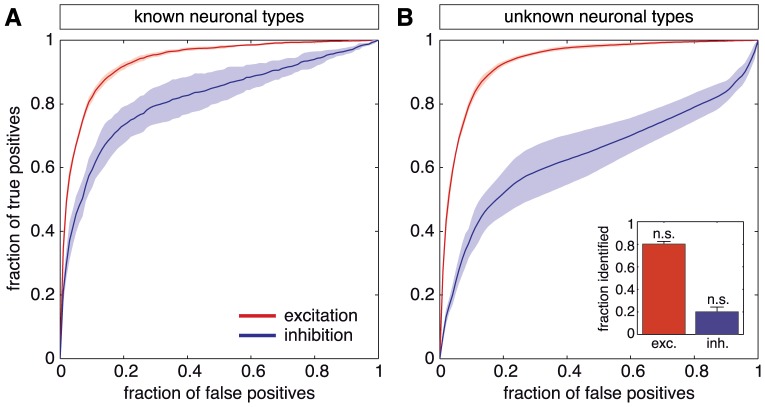
Optimal network reconstruction. **A** ROC curves for the reconstruction of a network with both excitatory and inhibitory connections active, supposing to know *a priori* information about neuronal type. GTE is first applied to the “E+I” data. Next, following Dale's principle and exploiting the available information on neuronal type, links are classified according to their excitatory (red) or inhibitory (blue) nature. **B** ROC curves for the best possible identification of excitatory and inhibitory connections, when information on neuronal type is unaccessible. Excitatory links (red) are identified by adding together the Transfer Entropy scores of simulations run in “E–only” and “E+I” conditions, and later thresholding them. Inhibitory links (blue) are identified by computing the difference in Transfer Entropy scores between the runs with inhibition present and blocked. Inset: fraction of excitatory and inhibitory neurons correctly identified from these ROC curves. Results were not significantly different from random guess (see Methods). All the results were averaged over different network realizations. The shaded areas in the main plots, as well as the error bars in the inset, correspond to 95% confidence intervals.

Being able to identify the type of a neuron — even with perfect accuracy — does not guarantee *a priori* that excitatory and inhibitory links can be inferred equally well. On the contrary, different reconstruction performances have to be expected in general, since the interaction mechanism of excitatory links is inherently different from the inhibitory ones, the former promoting the activity of the target neuron, whereas the latter restrain it. We have tested the accuracy of this *ad hoc* approach through numerical simulations. GTE is applied to the “E+I” data, and the reconstruction quality is assessed separately for the connections originating from neurons of different types (see Methods). Non trivially, the results of this analysis indicate that both types of connections are reconstructed with high accuracy (see Figure 3A). At a fraction of 10% of false positives, excitatory links are detected at a true positive rate of 80%. Inhibitory links show a lesser but still high detection accuracy, of about 60% of true positives.

### Reconstructing and labeling connections from spontaneous dynamics

In the absence of information on neuronal types, an alternative approach consists in a direct combination of the reconstructions procured by the “E–only” and “E+I” data on the same neurons. By adding together the GTE scores from the two reconstructions we can assume that the higher scores come from links that show a high score in both reconstructions. This procedure is thus expected to highlight the pool of excitatory connections, since they are the only ones present in both network conditions. Similarly, we can subtract the “E–only” scores from the “E+I” ones. High scores will then now highlight those links that are present in the “E+I” but not in the “E–only” network, i.e. the pool of inhibitory connections.

The performance of this first two-step reconstruction approach is shown in Figure 3B. The reconstruction of excitatory connections has a quality as good as the one obtained with *a priori* knowledge of neuronal type based on extrinsic labeling (see Figure 3A). However, the performance markedly deteriorates for the reconstruction of inhibitory links, since only 40% of the inhibitory connections are correctly identified at 10% of false positives.

Note that an additional complication arises with the described two-steps pipeline. A given link might be attributed a combined score above the inclusion threshold, both when considering the sum *and* the difference of original GTE scores. In this case, the link would be labeled as “both excitatory and inhibitory”, a fact which is excluded by Dale's principle. Despite this problem, we might still try to combine the “E–only” and “E+I” reconstructions to infer the nature of each neuron. To test the accuracy of such identification we try to label neurons as excitatory or inhibitory based on a highly “pure” structural network reconstruction. To do so, we select a very high GTE threshold for link, in such a way that in the inferred subnetwork —including, correspondingly, very few links only— the fraction of false positives remains small (with a maximally tolerable ratio of 5%). We first sum and subtract “E–only” and “E+I” scores to obtain putative excitatory and inhibitory links, as just discussed. We next compute the output degrees of the neurons for each subnetwork, 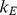 and 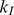, respectively. Finally, we rank each neuron according to the difference 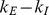. Following Dale's principle, the set of neurons with the highest (positive) ranking would be labeled as excitatory, and those with the lowest (negative) ranking as inhibitory. The results, however, as shown in the inset of Figure 3B, indicate that this approach does not provide better results than a random guessing of neuronal type (see Methods for details on significance testing) and a different approach is required.

### Reconstructing and labeling connections from stimulated dynamics

As a matter of fact, the major challenge for an accurate reconstruction and precise labeling of neuronal types is the identification of inhibitory links, and this for the following reason. To estimate GTE, we need to evaluate the probability of each given neuron to be active in a short time window of a duration 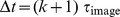, where 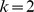 is the order of an assumed Markov approximation (see Methods) and 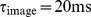 is the image acquisition interval. With these parameter choices, we obtain then 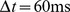. Neurons in a culture spike with an average inter-burst frequency of 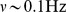, resulting in a low firing probability within each time bin. Continuing this reasoning, the probability that two unconnected neurons spike at random in the same time window is given by 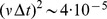. The number of coinciding events 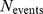 expected in a recording is thus:
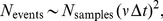
(1)where 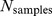 is the number of independent samples in a recording. In a typical recording session lasting 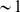 h, one gets 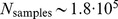 independent samples and therefore 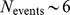. Hence, one can expect to observe, on average, just six concurrent spikes between any pair of unconnected neurons. If an excitatory link exists between two neurons, the conditional probability of firing rises above this random level and more coincidence events are observed, turning into an appreciable contribution to the GTE calculation. However, if an inhibitory link is present, the number of simultaneous spikes gets further reduced with respect to the already very small chance level, making any accurate statistical assessment very difficult. Nevertheless, we note that the number of detected events scales as 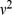 with the frequency of firing, and even a slight increase in spiking frequency would enhance considerably the reconstruction performance.

A promising approach to increase neuronal firing consists in forcing the neuronal network through external stimulation. Several studies on neuronal cultures have used external drives, typically in the form of electrical stimulation, to act on neuronal network activity, for instance to investigate connectivity traits [51], [62], modify or control activity patterns [63], [64], or explore network plasticity [65], [66]. Such *in vitro* approaches are reminiscent of *in vivo* clinically relevant techniques such as deep brain stimulation, used in the treatment of epilepsy and movement disorders [67], [68].

External stimulation in neuronal cultures has been reported to increase neuronal firing [64] and to reduce network bursting [63], [65], a combination of factors that, in the GTE reconstruction context, improve the accuracy in the identification of the network architecture. To explore potential improvements in reconstruction, we simulate the effect of an applied external drive in a purely phenomenological way by increasing the frequency parameter of the Poisson process that drives spontaneous activity. This additional drive never increases the spontaneous firing frequency beyond 3 Hz, being meant to represent the effects of a rather weak external stimulation. Due to this contained increase of firing rate, the collective bursting activity of the simulated network continues to be shaped dominantly by network interactions, rather than by the drive itself.

**Figure 4:**
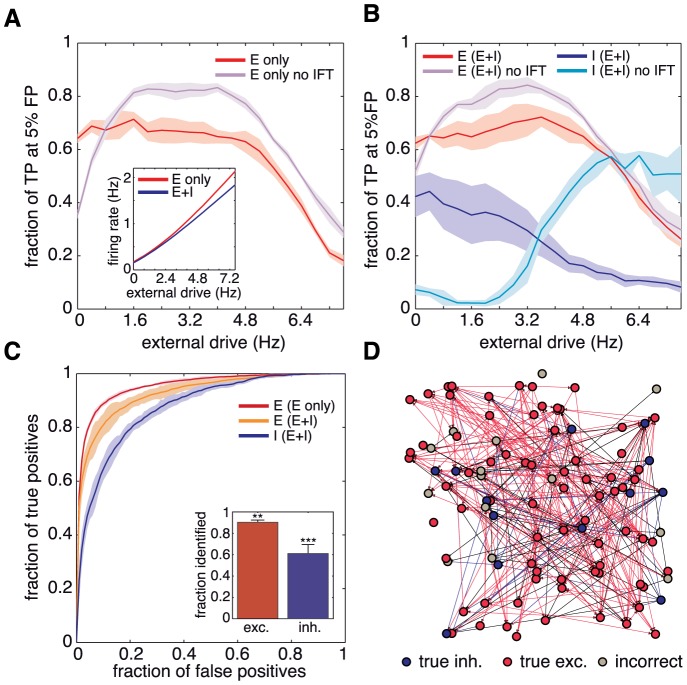
Reconstruction improvement through external stimulation. **A** and **B**, fraction of true positives from the reconstructions at the 5% false positive mark for the studied networks. “E–only” networks are shown in **A**; “E+I” networks in **B**. Inset: dependence of the spontaneous firing rate on the applied external drive, emulated here by increasing the rate of the background drive to the culture *in silico*. All the excitatory reconstructions reach a stable plateau in the reconstruction after removal of the instantaneous feedback term (IFT) correction (see Methods). The inhibitory reconstruction is accurate only for higher values of the external drive. **C** ROC curves extracted from **A** and **B** with an external stimulation of 4 Hz. Inset: fraction of excitatory and inhibitory neurons correctly identified from these reconstructions. Identification was statistically significant compared to random guessing. For excitatory neurons, 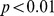 (**); for inhibitory neurons, 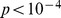 (***). **D** Example of an actual reconstruction after identification of neuronal type. Identified excitatory neurons are shown in red and inhibitory ones in blue. Incorrectly identified neurons are shown in grey. Correctly identified excitatory and inhibitory links are shown in red and blue, respectively, and wrongly identified links are shown in black. For clarity in the representation of the links, a threshold value lower than the optimal has been applied.

The performance of our GTE algorithm combined with a weak network stimulation is illustrated in Fig. 4A, where we show the fraction of true positives in the reconstruction of “E–only” networks at 5% false positives. The presence of even very small external drives substantially enhances reconstruction based on GTE. For higher drives, reconstruction performance reaches a plateau that quantifies the range of optimum stimulation. Performance later decays due to the excess of stimulation, which substantially perturbs spontaneous activity and alters qualitatively the global network dynamics. We incidentally remark that the incorporation of the external drive makes unnecessary — actually, even deleterious — the instantaneous feedback term correction (IFT, see Methods), i.e., an *ad hoc* modification to the original formulation of TE which was introduced in [22] to cope with the poor frame rate of calcium fluorescence recordings, definitely slower than the time-scale of monosynaptic interaction delays. The IFT correction allows to encompass interactions occurring in the same temporal bin of the recording for TE estimation, a feature useful to enhance reconstruction results when the time-scale of pre-postsynaptic neuron interactions is fast relative to the time resolution of the recording. However, same–bin interactions also result in an overestimation of bidirectional connections, since one cannot establish directionality within a single time bin. When the firing rate is enhanced with respect to spontaneous conditions these negative effects of the IFT corrections become predominant.

The same reconstruction analysis for “E+I” networks is shown in Fig. 4B, for excitatory and inhibitory links separately. The identification of excitatory links greatly improves with moderate drives and, again, IFT becomes unnecessary. For inhibitory links, performance is optimum at low drives, when IFT is used. Without IFT, however, performance is better at relatively high drives, and one can observe the existence of an optimal stimulation range (leading to a firing rate of 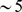 Hz) that maximizes inhibition reconstruction while preserving a relatively good excitatory identification.

We note as well that, for “E+I” networks, bursts disappear in general at higher values of the external drive. In general, as depicted in the inset of Fig. 4A, the dependence of the spontaneous firing frequency on the external drive is quantitatively different from “E–only” networks, requiring typically a stronger drive to achieve a comparable firing rate.

With the external drive the overall ROC curves are also improved. In Figure 4C we show the reconstruction performance for medium values of stimulation. In this new regime, we can again try to determine the neuronal type based on the labeling procedure used in the previous section (inset of Figure 4C). Now excitatory neurons are correctly identified with 90% accuracy, whereas the fraction of inhibitory neurons correctly identified rises conspicuously to 60%. This marked improvement is now statistically significant (see Methods).

In Figure 4D we show an actual reconstruction of a portion of the original network with this procedure. Correctly inferred excitatory and inhibitory neurons are shown in red and blue respectively, and mismatches in yellow. Correctly identified excitatory and inhibitory links are also shown in red and blue respectively, and false positives are shown in black. It is visually evident that for this thresholding level a very high purity is achieved, and only a small fraction of the reconstructed links are false positives.

We conclude that the addition of a weak external stimulation to the “bare” network dynamics results in an overall improvement on the reconstruction of both excitatory and inhibitory links. Moreover, by combining the reconstructions of “E–only” and “E+I” networks, we also become able to infer the neuronal type by just analyzing the dynamics, with no *a priori* knowledge of the system and without resorting to extrinsic information of any sort.

## Discussion

Living neuronal networks contain both excitatory and inhibitory neurons. Although the interplay between excitation and inhibition gives rise to the rich dynamical traits and operational modes of brain circuits, inhibition is often neglected when analyzing functional characteristics of neuronal circuits, mostly because of its difficult identification and treatment. In this work we have made a first step towards filling this gap, and introduced a new algorithmic approach to infer inhibitory synaptic interactions from multivariate activity time-series. In the framework of a realistically simulated neuronal network that mimics in a semi-quantitative way key features of the behavior of neuronal cultures, we applied Generalized Transfer Entropy (and Dale's principle) to identify excitatory as well as inhibitory connections and neurons.

In a previous work [22], we developed the GTE framework and applied it to extract topological information from the dynamics of purely excitatory networks, but left as an open question the treatment of inhibition. Here we have shown that GTE has the potential to be applied without substantial modifications to recordings relative to cultures with active inhibition (“E+I” cultures). This data is characterized by an irregular bursting dynamics with overall lower — but distinctly fluctuating — fluorescence amplitudes as well as higher bursting frequencies than purely excitatory (“E–only”) signals. In general, GTE provided an overall good reconstruction of the “E+I” simulated data, hinting at the robustness and general applicability of the algorithm. This is a highly non trivial achievement of the algorithm, given the profoundly different functional profile of inhibitory actions. The GTE reconstruction alone performed well in identifying the existence of links between pairs of neurons, however, it was not sufficient to resolve their excitatory or inhibitory nature. Yet, we provided evidence through numerical experiments that this additional goal could be fulfilled by retrieving *a priori* information about the types of different neurons (e.g. through immunostaining or selective fluorescent dyes), or by combining the reconstructions obtained from both “E+I” and “E–only” recordings from a same network (thus, again relying uniquely on time-series analysis).

When *a priori* information about the type of each neuron is available, Dale's principle proves to be, at least in our simulations, a solid yet simple approach that allows the identification of the major connectivity traits of the neuronal network. However, when applying Dale's principle to actual, living neuronal networks recordings (see later), one has to consider its possible limitations, like the existence of (rare) exceptions to it [69]. We also remark that, in a more realistic context, other types of *a priori* information beyond the nature of the neurons and their processes could be considered, like, e.g. information about their spatial distribution. Although in this work we have considered only purely random distance-independent topologies, neuronal cultures grow on a bi-dimensional domain, and excitatory connections are typically of shorter range than inhibitory ones. This kind of information could be integrated in the analysis of network models that include metric properties and accounts for spatial embedding (such as [27], [70], [71]), as well as different connectivity rules for the generation of excitatory and inhibitory sub-networks.

A systematic extrinsic labeling of neuronal types might be difficult to achieve in large culture experiments. When *a priori* information is unavailable, our results show that the combination of the reconstructions for “E–only” and “E+I” spontaneous activity data fails at identifying robustly the inhibitory interactions. Nevertheless, we find that the reconstruction performance of excitatory links remains almost unchanged when inhibition is present, despite the fact that inhibition may substantially alter excitatory interactions, and in turn network dynamics, for instance through feedback and feedforward inhibitory loops. The observation that excitatory links are still correctly reconstructed in “E+I” data shows the robustness of the algorithm to the presence of different interactions in the system. We remark that the main factor determining the poor identification of inhibitory links is the weak firing rate during inter-burst epochs. Since, in a nearly asynchronous regime of inter-burst firing, the action of a direct inhibitory link manifests itself by reducing below the already small chance level the probability of firing coincidence between the two connected neurons, the recording of a larger amount of inhibitory firing would be required to improve the reconstruction of inhibitory couplings. Although the recording duration can be increased at will in numeric simulations, this is not the case for real experimental recordings, to which our algorithm aims at being applied.

In our simulations, we naturally achieved to increase single neuronal firing activity, and therefore reconstruction statistics through a weak external stimulation of the network, with neither a significant disturbance in neuronal network dynamics nor the need for substantially longer recordings. In many previous works resorting to external drives to stimulate network activity, both experimental and theoretical, the applied stimulation was supra-threshold, i.e. the stimulation triggered directly neuronal firing [51], [54], [62], [72], [73]. Our approach raises instead network excitability by a weak external drive that effectively increases activity without modifying the network intrinsic behavior, in the direction of other experimental studies that stimulated multiple sites of a neuronal culture via a multi-electrodes array, to either increase network firing, reduce the occurrence of bursting episodes, or investigate plasticity [64], [66]. Interestingly, these works observed that a weak stimulation along few hours did not induce plastic effects, i.e. did not change network behavior, thus making our reconstruction strategy of immediate applicability in experimental recordings.

In the present work we have exhibited experimental data only for qualitative comparison with fluorescence traces obtained from the numerical model. The experimental data could be analyzed in principle without need of any modification to the GTE formulation, but we found our present knowledge of the experimental recordings insufficient to get reliable reconstructions. In particular, we are lacking good estimates of the neuronal firing rate during the inter-burst periods, as well as the amount of fluorescence change caused by an action potential. The former does not allow to determine whether we expect enough events to make the reconstruction of inhibitory links feasible (see Eq. (1)), while the latter prevents the application of an optimal data discretization strategy that would reduce the minimal recording length needed for accurate results. Our study intends therefore to foster the future application of the workaround strategies here explored in experiments *in silico*, i.e., most notably: (i) a weak external stimulation to increase spontaneous activity; and (ii) the extrinsic labeling of excitatory and inhibitory neuronal cell bodies after the recording (to provide at least a partial source of *a priori* information) to be used in synergy with our algorithmic approach.

Finally, our reconstruction algorithm has the potential to be immediately applied to the analysis of fluorescence data in experimental recordings that are not affected by the aforementioned limitations. In particular, *in vivo* recordings and brain slice measurements [74]–[76] display a much richer activity at the individual neuron level than in the *in vitro* counterparts. Recent works have highlighted the ability of high speed multi-neuron calcium imaging to access neuronal circuits *in vivo* [77]–[79]. Our methodology can thus be directly applied to these data, particularly in those investigations that target the role of inhibition [80], [81], although systematic verification of the inferred connectivity (in absence of a known ground-truth structure) remains currently out of reach and validation is only possible at the statistical level.

## Methods

All procedures were approved by the Ethical Committee for Animal Experimentation of the University of Barcelona, under order DMAH-5461.

### Calcium traces from *in vitro* cultures

Experimental traces of fluorescence calcium signals were obtained from rat cortical cultures at day *in vitro* 12, following the procedures described in our previous work [22] and in other studies [27], [51], [82]. Briefly, rat cortical neurons from 18–19-day-old Sprague-Dawley embryos were dissected, dissociated and cultured on glass coverslips previously coated with poly–l–lysine. Cultures were incubated at 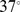
C, 95% humidity, and 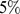 CO_2_. Each culture gave rise to a highly connected network within days that contained on the order of 500 neurons/mm^2^. Sustained spontaneous bursting activity appeared by day *in vitro* 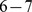. Prior to imaging, cultures were incubated for 40 min in recording medium containing the cell–permeant calcium sensitive dye Fluo-4-AM. The culture was washed with fresh medium after incubation and finally placed in a recording chamber for observation. The recording chamber was mounted on a Zeiss inverted microscope equipped with a Hamamatsu Orca Flash 2.8 CMOS camera. Fluorescence images were acquired with a speed of 50 frames per second and a spatial resolution of 3.4* µ*m/pixel.

In a typical measurement, we first recorded spontaneous activity as a long image sequence 60 min long. Both excitatory and inhibitory synapses were active in this first measurement (“E+I” network). We next fully blocked inhibitory synapses with 40* µ*M bicuculline, a GABA_A_ antagonist, so that activity was solely driven by excitatory neurons (“E–only” network). Activity was next measured again for another 60 min. At the end of the measurements, images were analyzed to to retrieve the evolution of the fluorescence signal for each neuron as a function of time.

Note once again that, in this study, experimental fluorescence traces were used only as a guiding reference for the design of synthetic data in “E–only' and “E+I” conditions, and were not analyzed to provide network reconstructions, given the limitations of current experimental protocols, highlighted in the Results and Discussion section.

### *In silico* model

#### Network generation

We randomly distributed 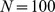 neurons over a square area of 1 mm^2^. Neurons were labeled as either excitatory with probability 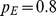 or inhibitory with 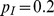. A directed connection (link) was created between any pair of neurons with fixed probability 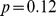, giving rise to a directed Erdős-Rényi network[83]. The resulting network is defined by the adjacency matrix 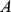, whose entries 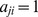 denote a connection from neuron 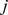 to neuron 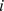 (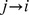). The average full clustering coefficient of the network [84] is given by
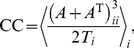
(2)where 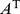 is the transpose of 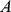 and 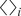 denotes average over index 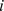. 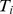 is defined as
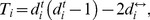
(3)where 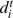 is the total degree of node 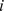 (the sum of its in– and out–degree) and 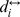 is the number of bidirectional links of node 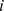. The clustering coefficient of the network after its construction was 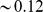, a value that was then raised up to a target one of 0.5 by following the Bansal *et al.* construction [85], as follows. Two existing links 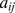 and 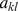 were first chosen at random, with 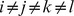. These links were then replaced by 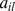 and 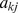. This step was repeated until the desired clustering coefficient was finally reached within a tolerance of 0.1%.

This above-chance clustering level was generated to account for experimental observations of clustered connections in neuronal local circuits [86]. We do not perform here a systematic study of the impact of CC on reconstruction performance, referring the reader to Ref. [22] for this issue, in which CC-independent performance is demonstrated.

#### Network dynamics

Neurons in the simulated culture were modeled as integrate-and-fire units, of the form
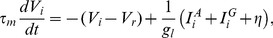
(4)where 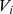 is 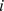-th neuron's membrane potential and 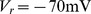 its resting value, 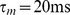 is the membrane time constant, 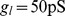 is the leak conductance, 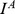 and 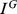 the excitatory (AMPA) and inhibitory (GABA_A_) input currents respectively, and 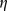 a noise term. When the membrane potential reaches the threshold value 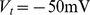 the neuron fires and its membrane potential is reset to a value 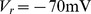, which is maintained for a refractory time 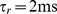 during which the neuron is prevented from firing.

Neurotransmitters were released as a response to a presynaptic action potential fired at time 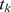, binding to the corresponding receptors at the postsynaptic side of its output neurons. The binding of neurotransmitters at the receptors triggered the generation of postsynaptic currents 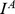 or 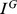, depending on the presynaptic neuronal type. The total input current received by a given neuron was described by
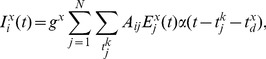
(5)where 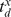 is a transmission delay (mimicking axonal conduction), with 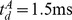 and 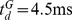. 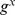 is the synaptic strength, which was adjusted to obtain the desired burst rate. The value of 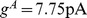 in a network with inhibition silenced provided a bursting rate of 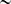
0.1 Hz. When inhibition was active, a comparable bursting rate of of 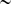
0.12 Hz was obtained by setting 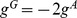. 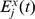 is a term accounting for short–term synaptic depression, and 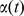 is an alpha shaped function of the form
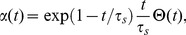
(6)where 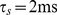 represents the synaptic rise time and 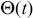 is the Heaviside step function.

Short–term synaptic depression accounts for the depletion of available neurotransmitters at the presynaptic terminals due to repeated activity [87]. The neurotransmitters dynamics at the synapses of neuron 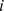 was described by the set of equations [88]:
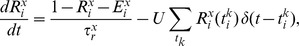


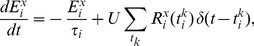
(7)where 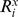 and 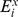 are the fraction of available neurotransmitters in the recovered and active states, respectively. 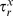 is the characteristic recovery time with 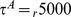 ms and 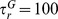 ms. 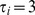 ms is the inactivation time and 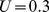 describes the fraction of activated synaptic resources after an action potential.

#### Simulating calcium fluorescence signals

Based on the simulated spike data, synthetic calcium fluorescence signals were generated according to a model that incorporates the calcium dynamics in the neurons and experimental artifacts. The former describes the saturating nature of calcium concentration bound to the calcium dye inside the cells, while the latter treats the noise of the recording camera as well as light scattering due to anisotropies in the recording medium [22].

Each action potential of a neuron 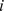 at time 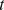 leads to the intake of 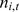 calcium ions through the cell membrane, raising the calcium concentration inside the cell. A number 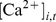 of the Calcium ions bind the fluorescence dye by a fixed amount 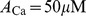, and are slowly freed with a time scale 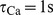. This process is described by the equation
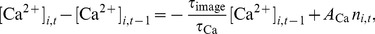
(8)where 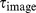 is the simulated image acquisition frame rate.

The level of calcium fluorescence 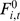 emitted by a cell was modeled by a Hill function of the bound calcium concentration (with saturation level 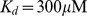) together with an additive Gaussian noise term 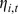 characterized with a standard deviation 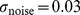 [59], i.e.
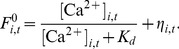
(9)


The level of fluorescence recorded by the camera at a given neuron was *not* independent of neighboring cells due to the introduction of simulated light scattering. We incorporated this artifact by adding to the monitored cell a fraction 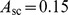 of the fluorescence from neighboring cells, which was weighted according to their mutual distance 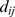 by a Gaussian kernel of width 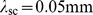. The total fluorescence captured in a neuron was then given by:
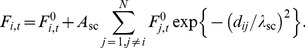
(10)


### Generalized Transfer Entropy

Generalized Transfer Entropy (GTE) was introduced in [22] as an extension of the original Transfer Entropy notion [35]. It is given by the Kullback-Leibler divergence between two probabilistic transition models for a given time series 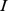, conditioned on the system visiting a specified target dynamical state. In the case of fluorescence signals, this state selection is achieved by conditioning the analysis to the regime where the population average of the time series 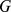 is lower than a given threshold 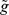, i.e.
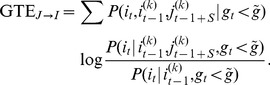
(11)


Here, vectors in time are denoted by their length in brackets, which is equal to the order of Markov order approximation assumed for the underlying process, 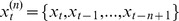. The sum is defined over all possible values of 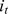 and the vectors 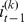 and 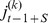. The shift variable 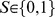 denotes the inclusion of same-bin (instantaneous) interactions for 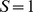. This adjustment was introduced in [22] to cope with the limited time-resolution of calcium fluorescence signals and is dubbed in the text as Instantaneous Feedback Term (IFT) correction. Furthermore, the time-series of calcium fluorescence were high-pass filtered by mean of a discrete difference operator, as a straightforward attempt to enhance the visibility of firing events drowned in noise. Note that GTE reduces to conventional Transfer Entropy for 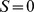 and 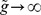, i.e. when same-bin interactions are excluded and when the selected state encompasses the whole observed dynamics. The Markov order of the underlying process is here somewhat arbitrarily set to 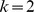, following on [22] where we extensively checked its effect on the reconstructions: in our previous study, 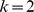 resulted to be the lowest dimensionality in the probability distribution allowing to separate actual interactions from signal artifacts like light scattering.

Note that we did not perform any delay embedding of the time-series, because we did not find it here necessary to reach satisfying performance levels, or leading to noticeable improvements. Methodological developments along the lines of [38], [39] would be however desirable for future applications to real experimental data.

Code for our Generalized Transfer Entropy method is publicly available at https://github.com/olavolav/TE-Causality.

### Optimal binning

The probability distributions in GTE as defined in Eq. (11) were estimated based on discretized values of the temporal difference signal of the observed fluorescence. To cope with potential undersampling artifacts —since the probability distributions to estimate have an elevated dimensionality, as large as 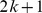
— we symbolized the signals into a binary sequence, by applying a sharp threshold. The optimal threshold value 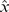 for this conversion was obtained from the following analysis. We first ignored the exponential decay of the fluorescence signal since it has a small influence on discretely differentiated signals, and assumed a sufficiently low firing rate so that the occurrence of more than one spike per frame of a given neuron is negligible. Under these simplification hypotheses, the probability distribution of the signal can be cast as a combination of Gaussian functions, with mean values given by the offset associated to the number of action potentials encountered in the current time bin. Additionally, to preserve information about spiking events when projecting the time-series into a binary representation, we computed the optimal mapping by determining the probability 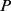 that the mapping is correct at any given time step (provided the parameters of the model 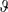 and a threshold value 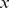), i.e.:
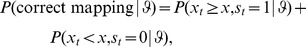
where 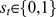 denotes the occurrence of a firing event at time frame 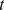, and 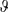 refers to unspecified but frozen parameters of the analyzed system, which have a potential influence on the estimated probability. In particular, the probability that a neuron fires at a given image frame is a function of the firing rate and the length of the image frame, 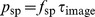. For a normally distributed camera noise with standard deviation 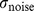 and an expected variation 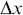 in fluorescence due to a single spike, a straightforward solution for the optimal separation value 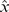 that yields the maximum of the correct mapping probability can be derived:



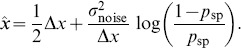
(12)GTE scores were robust against the selection of a separation value above the optimal 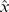. Indeed, for 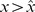 the total number of samples above the separating value is reduced, but the fraction of samples that correspond to real spikes is actually increased. The resulting network reconstructions did not show any notable decrease of quality for values of 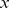 up to a 30% above the optimal value.

### Network reconstruction

In order to reconstruct a whole network, GTE was computed for each directed pair of neurons 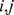 from Eq. (11), resulting in a matrix 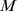 of directed causal influences where 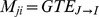. A new binary matrix 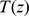 was created from the GTE scores, where 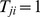 if 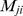 is amongst the fraction 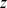 of links with the highest GTE score.

The quality of the reconstruction was quantified through a Receiver Operating Characteristic (ROC) analysis. The ROC is a parametric curve that establishes a relationship between the true and the false positive links found in 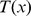 for the different thresholded levels. If 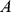 denotes the binary connectivity matrix of the real network, then the true positive ratio (TPR) is defined as the number of links in 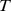 that are present in 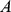 respect to the total number of existing links. The false positive ratio (FPR) is the fraction of links in 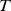 that do not match original links, i.e.,
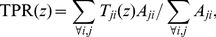
(13)

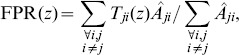
(14)where 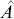 is the negation of the binary connectivity matrix 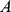 (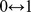). Thus 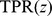 and 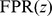 constitute, respectively, finite-size estimates of the probabilities 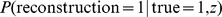 and 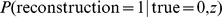, for any given link across the network. Confidence intervals for ROC curves were estimated based on 5 different network realizations.

#### Combining two reconstruction results

To distinguish between excitatory and inhibitory neurons, we combined the information of the reconstructions obtained from the “E+I” and “E–only” data, namely 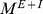 and 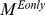. We assumed that excitatory links are present in both datasets, while inhibitory ones appear only in the “E+I” reconstruction, and proceeded by defining new matrices of putative excitatory 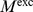 and putative inhibitory influences 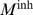, of the form:
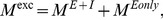
(15)

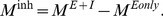
(16)


To obtain the effective connectivity reconstruction only the rank ordering of GTE values is relevant. Therefore no rescaling of these matrices is necessary, and the final set of links could be obtained by thresholding the matrices as described above.

To label the neurons as either excitatory or inhibitory, we first removed all links that were present in both reconstructions, and then ranked the neurons according to the difference between excitatory and inhibitory links, 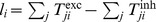. We next used the prior information that a fraction 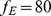
% of the neuronal population is excitatory, therefore identifying as excitatory neurons the 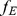 fraction with the highest 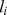 score, and labeling the rest as inhibitory.

#### Statistical tests

Statistical significance on the inference of excitatory and inhibitory neuronal types was performed as follows. Assuming that the fraction of excitatory and inhibitory neurons (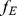 and 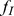 respectively) is known with good precision in a population of 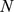 cells, the probability to correctly identify by chance a given set of neurons 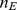 and 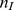 in a given trial 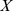 follows a binomial distribution:
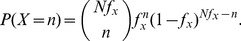
(17)


Let suppose that a labeling method provides a fraction 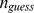 of correctly labeled links. We considered this labeling result as statistically significant if the probability of outperforming by chance this success rate was 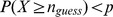, with a standard choice of 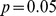.

## References

[1] Bullmore E, Sporns O (2009) Complex brain networks: Graph theoretical analysis of structural and functional systems. Nat Rev Neurosci 10: 186–198. 19190637 10.1038/nrn2575

[2] Power JD, Cohen AL, Nelson SM, Wig GS, Barnes KA, et al (2011) Functional network organization of the human brain. Neuron 72: 665–678. 22099467 10.1016/j.neuron.2011.09.006 PMC3222858

[3] Chicurel M (2000) Databasing the brain. Nature 406: 822–825. 10972265 10.1038/35022659

[4] Abbott A (2013) Neuroscience: Solving the brain. Nature 499: 272–274. 23868244 10.1038/499272a

[5] Alivisatos AP, Chun M, Church GM, Greenspan RJ, Roukes ML, et al (2012) The brain activity map project and the challenge of functional connectomics. Neuron 74: 970–974. 22726828 10.1016/j.neuron.2012.06.006 PMC3597383

[6] Alivisatos AP, Chun M, Church GM, Deisseroth K, Donoghue JP, et al (2013) The brain activity map. Science 339: 1284–1285. 23470729 10.1126/science.1236939 PMC3722427

[7] Hagmann P, Cammoun L, Gigandet X, Meuli R, Honey CJ, et al (2008) Mapping the structural core of human cerebral cortex. PLoS Biol 6: e159. 18597554 10.1371/journal.pbio.0060159 PMC2443193

[8] Deco G, Jirsa VK, McIntosh AR (2011) Emerging concepts for the dynamical organization of resting-state activity in the brain. Nat Rev Neurosci 12: 43–56. 21170073 10.1038/nrn2961

[9] Varela F, Lachaux JP, Rodriguez E, Martinerie J (2001) The brainweb: Phase synchronization and large-scale integration. Nat Rev Neurosci 2: 229–239. 11283746 10.1038/35067550

[10] Raizada RDS, Grossberg S (2003) Towards a theory of the laminar architecture of cerebral cortex: Computational clues from the visual system. Cereb Cortex 13: 100–113. 12466221 10.1093/cercor/13.1.100

[11] Corbetta M, Patel G, Shulman GL (2008) The reorienting system of the human brain: From environment to theory of mind. Neuron 58: 306–324. 18466742 10.1016/j.neuron.2008.04.017 PMC2441869

[12] Gaillard R, Dehaene S, Adam C, Clémenceau S, Hasboun D, et al (2009) Converging intracranial markers of conscious access. PLoS Biol 7: e61. 19296722 10.1371/journal.pbio.1000061 PMC2656551

[13] Zhou J, Gennatas ED, Kramer JH, Miller BL, Seeley WW (2012) Predicting regional neurodegeneration from the healthy brain functional connectome. Neuron 73: 1216–1227. 22445348 10.1016/j.neuron.2012.03.004 PMC3361461

[14] Seeley WW, Crawford RK, Zhou J, Miller BL, Greicius MD (2009) Neurodegenerative diseases target large-scale human brain networks. Neuron 62: 42–52. 19376066 10.1016/j.neuron.2009.03.024 PMC2691647

[15] Lynall ME, Bassett DS, Kerwin R, McKenna PJ, Kitzbichler M, et al (2010) Functional connectivity and brain networks in schizophrenia. J Neurosci 30: 9477–9487. 20631176 10.1523/JNEUROSCI.0333-10.2010 PMC2914251

[16] Bock DD, Lee WCA, Kerlin AM, Andermann ML, Hood G, et al (2011) Network anatomy and in vivo physiology of visual cortical neurons. Nature 471: 177–182. 21390124 10.1038/nature09802 PMC3095821

[17] Friston KJ (2011) Functional and Effective Connectivity: A Review. Brain Connect 1: 13–36. 22432952 10.1089/brain.2011.0008

[18] Wang Z, Chen LM, Ngyessy L, Friedman RM, Mishra A, et al (2013) The relationship of anatomical and functional connectivity to resting-state connectivity in primate somatosensory cortex. Neuron 78: 1116–1126. 23791200 10.1016/j.neuron.2013.04.023 PMC3723346

[19] Honey CJ, Sporns O, Cammoun L, Gigandet X, Thiran JP, et al (2009) Predicting human restingstate functional connectivity from structural connectivity. Proc Natl Acad Sci U S A 106: 2035–2040. 19188601 10.1073/pnas.0811168106 PMC2634800

[20] Battaglia D, Witt A, Wolf F, Geisel T (2012) Dynamic effective connectivity of inter-areal brain circuits. PLoS Comput Biol 8: e1002438. 22457614 10.1371/journal.pcbi.1002438 PMC3310731

[21] Deco G, Jirsa VK (2012) Ongoing cortical activity at rest: criticality, multistability, and ghost attractors. J Neurosci 32: 3366–3375. 22399758 10.1523/JNEUROSCI.2523-11.2012 PMC6621046

[22] Stetter O, Battaglia D, Soriano J, Geisel T (2012) Model-Free Reconstruction of Excitatory Neuronal Connectivity from Calcium Imaging Signals. PLoS Comput Biol 8: e1002653. 22927808 10.1371/journal.pcbi.1002653 PMC3426566

[23] Logothetis NK (2008) What we can do and what we cannot do with fMRI. Nature 453: 869–878. 18548064 10.1038/nature06976

[24] Eckmann JP, Feinerman O, Gruendlinger L, Moses E, Soriano J, et al (2007) The physics of living neural networks. Phys Rep 449: 54–76.

[25] Wheeler B, Brewer G (2010) Designing neural networks in culture. Proc IEEE 98: 398–406. 10.1109/JPROC.2009.2039029 PMC3101502 21625406

[26] Maeda E, Robinson HP, Kawana A (1995) The mechanisms of generation and propagation of synchronized bursting in developing networks of cortical neurons. J Neurosci 15: 6834–6845. 7472441 10.1523/JNEUROSCI.15-10-06834.1995 PMC6578010

[27] Orlandi JG, Soriano J, Alvarez-Lacalle E, Teller S, Casademunt J (2013) Noise focusing and the emergence of coherent activity in neuronal cultures. Nat Phys 9: 582–590.

[28] Eytan D, Marom S (2006) Dynamics and effective topology underlying synchronization in networks of cortical neurons. J Neurosci 26: 8465–8476. 16914671 10.1523/JNEUROSCI.1627-06.2006 PMC6674346

[29] Wagenaar DA, Pine J, Potter SM (2006) An extremely rich repertoire of bursting patterns during the development of cortical cultures. BMC Neurosci 7: 11. 16464257 10.1186/1471-2202-7-11 PMC1420316

[30] Cohen E, Ivenshitz M, Amor-Baroukh V, Greenberger V, Segal M (2008) Determinants of spontaneous activity in networks of cultured hippocampus. Brain Res 1235: 21–30. 18602907 10.1016/j.brainres.2008.06.022

[31] Pasquale V, Massobrio P, Bologna L, Chiappalone M, Martinoia S (2008) Self-organization and neuronal avalanches in networks of dissociated cortical neurons. Neurosci 153: 1354–1369. 10.1016/j.neuroscience.2008.03.050 18448256

[32] Tetzlaff C, Okujeni S, Egert U, Wörgötter F, Butz M (2010) Self-organized criticality in developing neuronal networks. PLoS Comput Biol 6: e1001013. 21152008 10.1371/journal.pcbi.1001013 PMC2996321

[33] Mazzoni A, Broccard FD, Garcia-Perez E, Bonifazi P, Ruaro ME, et al (2007) On the dynamics of the spontaneous activity in neuronal networks. PLoS ONE 2: e439. 17502919 10.1371/journal.pone.0000439 PMC1857824

[34] Kaiser A, Schreiber T (2002) Information transfer in continuous processes. Physica D 166: 43–62.

[35] Schreiber T (2000) Measuring information transfer. Phys Rev Lett 85: 461–464. 10991308 10.1103/PhysRevLett.85.461

[36] Gourévitch B, Eggermont JJ (2007) Evaluating information transfer between auditory cortical neurons. J Neurophysiol 97: 2533–2543. 17202243 10.1152/jn.01106.2006

[37] Besserve M, Schölkopf B, Logothetis NK, Panzeri S (2010) Causal relationships between frequency bands of extracellular signals in visual cortex revealed by an information theoretic analysis. J Comput Neurosci 29: 547–566. 20396940 10.1007/s10827-010-0236-5 PMC2978901

[38] Wibral M, Rahm B, Rieder M, Lindner M, Vicente R, et al (2011) Transfer entropy in magnetoencephalographic data: Quantifying information flow in cortical and cerebellar networks. Prog Biophys Mol Biol 105: 80–97. 21115029 10.1016/j.pbiomolbio.2010.11.006

[39] Vicente R, Wibral M, Lindner M, Pipa G (2010) Transfer entropy: A model-free measure of effective connectivity for the neurosciences. J Comput Neurosci 30: 45–67. 20706781 10.1007/s10827-010-0262-3 PMC3040354

[40] Kobayashi R, Kitano K (2013) Impact of network topology on inference of synaptic connectivity from multi-neuronal spike data simulated by a large-scale cortical network model. J Comput Neurosci 35: 109–124. 23388860 10.1007/s10827-013-0443-y

[41] Bettencourt LMA, Stephens GJ, Ham MI, Gross GW (2007) Functional structure of cortical neuronal networks grown in vitro. Phys Rev E 75: 021915. 10.1103/PhysRevE.75.021915 17358375

[42] Garofalo M, Nieus T, Massobrio P, Martinoia S (2009) Evaluation of the performance of information theory-based methods and cross-correlation to estimate the functional connectivity in cortical networks. PLoS ONE 4: e6482. 19652720 10.1371/journal.pone.0006482 PMC2715865

[43] Ito S, Hansen ME, Heiland R, Lumsdaine A, Litke AM, et al (2011) Extending transfer entropy improves identification of effective connectivity in a spiking cortical network model. PLoS ONE 6: e27431. 22102894 10.1371/journal.pone.0027431 PMC3216957

[44] Marconi E, Nieus T, Maccione A, Valente P, Simi A, et al (2012) Emergent functional properties of neuronal networks with controlled topology. PLoS ONE 7: e34648. 22493706 10.1371/journal.pone.0034648 PMC3321036

[45] Poil SS, Hardstone R, Mansvelder HD, Linkenkaer-Hansen K (2012) Critical-state dynamics of avalanches and oscillations jointly emerge from balanced excitation/inhibition in neuronal networks. J Neurosci 32: 9817–9823. 22815496 10.1523/JNEUROSCI.5990-11.2012 PMC3553543

[46] Lombardi F, Herrmann HJ, Perrone-Capano C, Plenz D, de Arcangelis L (2012) Balance between excitation and inhibition controls the temporal organization of neuronal avalanches. Phys Rev Lett 108: 228703. 23003665 10.1103/PhysRevLett.108.228703

[47] Isaacson JS, Scanziani M (2011) How inhibition shapes cortical activity. Neuron 72: 231–243. 22017986 10.1016/j.neuron.2011.09.027 PMC3236361

[48] Haider B, Husser M, Carandini M (2012) Inhibition dominates sensory responses in the awake cortex. Nature 493: 97–100. 23172139 10.1038/nature11665 PMC3537822

[49] Arber S (2012) Motor circuits in action: Specification, connectivity, and function. Neuron 74: 975–989. 22726829 10.1016/j.neuron.2012.05.011

[50] Yizhar O, Fenno LE, Prigge M, Schneider F, Davidson TJ, et al (2011) Neocortical excitation/inhibition balance in information processing and social dysfunction. Nature 477: 171–178. 21796121 10.1038/nature10360 PMC4155501

[51] Soriano J, Rodríguez Martínez M, Tlusty T, Moses E (2008) Development of input connections in neural cultures. Proc Natl Acad Sci U S A 105: 13758–13763. 18772389 10.1073/pnas.0707492105 PMC2544527

[52] Chiappalone M, Bove M, Vato A, Tedesco M, Martinoia S (2006) Dissociated cortical networks show spontaneously correlated activity patterns during in vitro development. Brain Res 1093: 41–53. 16712817 10.1016/j.brainres.2006.03.049

[53] Grienberger C, Konnerth A (2012) Imaging calcium in neurons. Neuron 73: 862–885. 22405199 10.1016/j.neuron.2012.02.011

[54] Jacobi S, Soriano J, Segal M, Moses E (2009) BDNF and NT-3 increase excitatory input connectivity in rat hippocampal cultures. Eur J Neurosci 30: 998–1010. 19723292 10.1111/j.1460-9568.2009.06891.x

[55] Tibau E, Valencia M, Soriano J (2013) Identification of neuronal network properties from the spectral analysis of calcium imaging signals in neuronal cultures. Front Neural Circuits 7: 199. 24385953 10.3389/fncir.2013.00199 PMC3866384

[56] Cohen D, Segal M (2011) Network bursts in hippocampal microcultures are terminated by exhaustion of vesicle pools. J Neurophysiol 106: 2314–2321. 21832037 10.1152/jn.00969.2010

[57] Granger C (1969) Investigating Causal Relations by Econometric Models and Cross-spectral Methods. Econometrica 37: 424–438.

[58] Pajevic S, Plenz D (2009) Efficient network reconstruction from dynamical cascades identifies small-world topology of neuronal avalanches. PLoS Computational Biology 5: e1000271. 19180180 10.1371/journal.pcbi.1000271 PMC2615076

[59] Mishchenko Y, Vogelstein JT, Paninski L (2011) A Bayesian approach for inferring neuronal connectivity from calcium uorescent imaging data. Ann Appl Stat 5: 1229–1261.

[60] Lichtman JW, Livet J, Sanes JR (2008) A technicolour approach to the connectome. Nat Rev Neurosci 9: 417–422. 18446160 10.1038/nrn2391 PMC2577038

[61] Kandel E (1967) Dale's principle and the functional specificity of neurons. Electrophys Stud Neuropharmacol Kolle, W(ed) Springfield, Ill: CC Thomas: 385–398.

[62] Breskin I, Soriano J, Moses E, Tlusty T (2006) Percolation in living neural networks. Phys Rev Lett 97: 188102. 17155581 10.1103/PhysRevLett.97.188102

[63] Wagenaar DA, Madhavan R, Pine J, Potter SM (2005) Controlling bursting in cortical cultures with closed-loop multi-electrode stimulation. J Neurosci 25: 680–688. 15659605 10.1523/JNEUROSCI.4209-04.2005 PMC2663856

[64] van Pelt J, Vajda I, Wolters PS, Corner MA, Ramakers GJ (2005) Dynamics and plasticity in developing neuronal networks in vitro. Progr Brain Res 147: 171–188. 10.1016/S0079-6123(04)47013-7 15581705

[65] Madhavan R, Chao Z, Wagenaar D, Bakkum D, Potter S (2006) Multi-site stimulation quiets network-wide spontaneous bursts and enhances functional plasticity in cultured cortical networks. In: Engineering in Medicine and Biology Society, 2006. EMBS '06. 28th Annual International Conference of the IEEE. pp. 1593–1596. 10.1109/IEMBS.2006.260571 17946052

[66] Wagenaar D, Pine J, Potter S (2006) Searching for plasticity in dissociated cortical cultures on multi-electrode arrays. J Negat Results Biomed 5: 16. 17067395 10.1186/1477-5751-5-16 PMC1800351

[67] McIntyre CC, Savasta M, Goff LKL, Vitek JL (2004) Uncovering the mechanism(s) of action of deep brain stimulation: Activation, inhibition, or both. Clin Neurophysiol 115: 1239–1248. 15134690 10.1016/j.clinph.2003.12.024

[68] Durand D, Bikson M (2001) Suppression and control of epileptiform activity by electrical stimulation: a review. Proc IEEE 89: 1065–1082.

[69] Nicoll RA, Malenka RC (1998) A tale of two transmitters. Science 281: 360–361. 9705712 10.1126/science.281.5375.360

[70] Eckmann JP, Moses E, Stetter O, Tlusty T, Zbinden C (2010) Leaders of neuronal cultures in a quorum percolation model. Front Comput Neurosci 4. 10.3389/fncom.2010.00132 PMC2955434 20953239

[71] Schmeltzer C, Soriano J, Sokolov IM, Rüdiger S (2014) Percolation of spatially constrained Erdös-Rényi networks with degree correlations. Phys Rev E 89: 012116. 10.1103/PhysRevE.89.012116 24580181

[72] Cohen O, Keselman A, Moses E, Rodríguez Martínez M, Soriano J, et al (2010) Quorum percolation in living neural networks. Europhys Lett 89: 18008.

[73] Linaro D, Storace M, Mattia M (2011) Inferring network dynamics and neuron properties from population recordings. Front Comput Neurosci 5. 10.3389/fncom.2011.00043 PMC3191764 22016731

[74] Mao BQ, Hamzei-Sichani F, Aronov D, Froemke RC, Yuste R (2001) Dynamics of spontaneous activity in neocortical slices. Neuron 32: 883–898. 11738033 10.1016/s0896-6273(01)00518-9

[75] Brustein E, Marandi N, Kovalchuk Y, Drapeau P, Konnerth A (2003) "in vivo" monitoring of neuronal network activity in zebrafish by two-photon Ca2+ imaging. Pflügers Archiv 446: 766–773. 12883893 10.1007/s00424-003-1138-4

[76] Dombeck DA, Khabbaz AN, Collman F, Adelman TL, Tank DW (2007) Imaging large-scale neural activity with cellular resolution in awake, mobile mice. Neuron 56: 43–57. 17920014 10.1016/j.neuron.2007.08.003 PMC2268027

[77] Stosiek C, Garaschuk O, Holthoff K, Konnerth A (2003) In vivo two-photon calcium imaging of neuronal networks. Proc Natl Acad Sci U S A 100: 7319–7324. 12777621 10.1073/pnas.1232232100 PMC165873

[78] Kerr JN, Denk W (2008) Imaging in vivo: watching the brain in action. Nat Rev Neurosci 9: 195–205. 18270513 10.1038/nrn2338

[79] Grewe BF, Langer D, Kasper H, Kampa BM, Helmchen F (2010) High-speed in vivo calcium imaging reveals neuronal network activity with near-millisecond precision. Nat Methods 7: 399–405. 20400966 10.1038/nmeth.1453

[80] Bonifazi P, Goldin M, Picardo MA, Jorquera I, Cattani A, et al (2009) Gabaergic hub neurons orchestrate synchrony in developing hippocampal networks. Science 326: 1419–1424. 19965761 10.1126/science.1175509

[81] Marissal T, Bonifazi P, Picardo MA, Nardou R, Petit LF, et al (2012) Pioneer glutamatergic cells develop into a morpho-functionally distinct population in the juvenile ca3 hippocampus. Nat Commun 3: 1316. 23271650 10.1038/ncomms2318 PMC3535425

[82] Segal M, Manor D (1992) Confocal microscopic imaging of [Ca2+]i in cultured rat hippocampal neurons following exposure to N-methyl-D-aspartate. J Physiol 448: 655–676. 1534370 10.1113/jphysiol.1992.sp019063 PMC1176221

[83] Albert R, Barabási AL (2002) Statistical mechanics of complex networks. Rev Mod Phys 74: 47–97.

[84] Fagiolo G (2007) Clustering in complex directed networks. Phys Rev E 76: 26107. 10.1103/PhysRevE.76.026107 17930104

[85] Bansal S, Khandelwal S, Meyers LA (2009) Exploring biological network structure with clustered random networks. BMC Bioinformatics 10: 405. 20003212 10.1186/1471-2105-10-405 PMC2801686

[86] Perin R, Berger TK, Markram H (2011) A synaptic organizing principle for cortical neuronal groups. Proc Natl Acad Sci U S A 108: 5419–5424. 21383177 10.1073/pnas.1016051108 PMC3069183

[87] Zucker RS, Regehr WG (2002) Short-term synaptic plasticity. Annu Rev Physiol 64: 355–405. 11826273 10.1146/annurev.physiol.64.092501.114547

[88] Tsodyks MV, Markram H (1997) The neural code between neocortical pyramidal neurons depends on neurotransmitter release probability. Proc Natl Acad Sci U S A 94: 719–723. 9012851 10.1073/pnas.94.2.719 PMC19580

